# Functional Connectivity Estimated from Resting-State fMRI Reveals Selective Alterations in Male Adolescents with Pure Conduct Disorder

**DOI:** 10.1371/journal.pone.0145668

**Published:** 2015-12-29

**Authors:** Feng-Mei Lu, Jian-Song Zhou, Jiang Zhang, Yu-Tao Xiang, Jian Zhang, Qi Liu, Xiao-Ping Wang, Zhen Yuan

**Affiliations:** 1 Bioimaging Core, Faculty of Health Sciences, University of Macau, Taipa, Macau SAR, China; 2 Mental Health Institute, Second Xiangya Hospital, Central South University, Hunan Province Technology Institute of Psychiatry, Key Laboratory of Psychiatry and Mental Health of Hunan Province, Changsha, Hunan 410011, China; 3 Department of Medical Information Engineering, School of Electrical Engineering and Information, Sichuan University, Chengdu 610065, China; Peking University, CHINA

## Abstract

Conduct disorder (CD) is characterized by a persistent pattern of antisocial behavior and aggression in childhood and adolescence. Previous task-based and resting-state functional magnetic resonance imaging (fMRI) studies have revealed widespread brain regional abnormalities in adolescents with CD. However, whether the resting-state networks (RSNs) are altered in adolescents with CD remains unknown. In this study, resting-state fMRI data were first acquired from eighteen male adolescents with pure CD and eighteen age- and gender-matched typically developing (TD) individuals. Independent component analysis (ICA) was implemented to extract nine representative RSNs, and the generated RSNs were then compared to show the differences between the CD and TD groups. Interestingly, it was observed from the brain mapping results that compared with the TD group, the CD group manifested decreased functional connectivity in four representative RSNs: the anterior default mode network (left middle frontal gyrus), which is considered to be correlated with impaired social cognition, the somatosensory network (bilateral supplementary motor area and right postcentral gyrus), the lateral visual network (left superior occipital gyrus), and the medial visual network (right fusiform, left lingual gyrus and right calcarine), which are expected to be relevant to the perceptual systems responsible for perceptual dysfunction in male adolescents with CD. Importantly, the novel findings suggested that male adolescents with pure CD were identified to have dysfunctions in both low-level perceptual networks (the somatosensory network and visual network) and a high-order cognitive network (the default mode network). Revealing the changes in the functional connectivity of these RSNs enhances our understanding of the neural mechanisms underlying the modulation of emotion and social cognition and the regulation of perception in adolescents with CD.

## Introduction

Conduct disorder (CD) is described as a disorder involving the violation of the basic rights of others and societal rules combined with a pervasive pattern of antisocial and aggressive behaviors (DSM-5, Diagnostic and Statistical Manual of Mental Disorders) [1]. In particular, adolescents with CD will engage in disruptive behaviors that not only cause a serious physical harm to other people but also present a high risk of developing various psychopathologic conditions, such as substance abuse, major depression, antisocial personality disorder and suicide [2]. Recent work using task-based or resting-state functional magnetic resonance imaging (fMRI) has suggested that brain regions, including the insula [3–5], anterior cingulate cortex [4], amygdala [5, 6], orbitofrontal cortex [7], caudate nucleus [7, 8], bilateral temporal-parietal regions [9], and fusiform gyrus [3], are strongly associated with functional impairments among adolescents with CD. However, exploring the functional abnormalities by mapping sites of cortical changes is insufficient to explain the pathogenesis of CD in adolescents. In particular, the alterations of the brain’s neuronal circuit that connects these sites is widely recognized to be related to CD. As such, further investigations at the brain network level are essential to identify the altered connectivity among various brain regions to disentangle the neural mechanism underlying CD in adolescents.

In recent decades, resting-state functional connectivity MRI (rs-fcMRI) has received extensive attention due to its unique advantages in mapping brain networks, characterizing brain functional changes among different patients and healthy controls, and analyzing the cognitive processes. In rs-fcMRI, low-frequency (0.01–0.1 Hz) fluctuations in the blood oxygen level-dependent (BOLD) fMRI signal reflect spontaneous neural activity during the resting state [10, 11]. In particular, rs-fcMRI has been utilized to assess the disrupted functional connectivity in different neuropsychiatric disorders [12], such as Alzheimer's disease [13], depression [14], schizophrenia [15], social anxiety disorder [16] and epilepsy [17]. Interestingly, these brain disruptions are related to the alterations of coherent intrinsic neuronal activity reflected by spontaneous BOLD fluctuations. Recent functional connectivity studies have demonstrated that intrinsic brain activity during the resting state is organized into several anatomically segregated but functionally correlated networks, known as resting-state networks (RSNs) [18, 19]. Importantly, such RSNs are able to characterize the neural mechanisms associated with somato-motor, visual, auditory, dorsal attention, memory, language, and default mode networks, which are generally modulated during task performances [18–27]. To identify the RSNs that are correlated with psychiatric or neurological disorders, the seed-based correlation method [28, 29] and independent component analysis (ICA) [30–32] are the most common approaches used to assess the functional connectivity. The seed-based method is a hypothesis-driven approach, which requires *a priori* knowledge of a region of interest (ROI) to quantify the temporal correlation between the ROI (a seed) and other brain regions [33]. In contrast, ICA is a data-driven based method and is able to generate spatiotemporal components in terms of blind source separation and linear decomposition of fMRI data. ICA does not require one to define an *a priori* time course or a specific seed region, thus making it an ideal choice for constructing RSNs [30].

In addition, functional neuroimaging evidence accumulated in recent years has suggested that the cognitive and emotional dysfunctions in adolescent CD were strongly associated with widespread regional deficits [6, 9, 34]. In this study, we hypothesized that functional connectivity in RSNs should be different between the adolescents with CD group and the typically-developing (TD) group and that the alterations in the brain’s neuronal circuit should have a powerful influence on the brain organization of adolescents with CD. In particular, rs-fcMRI was utilized to identify the distributed brain networks to better understand the neural mechanisms in adolescents with pure CD. The RSNs were generated by group spatial ICA, which was conducted for all the participants, including adolescent CDs and TD subjects and were then compared between the two groups to explore distinct differences in the functional connectivity. It was noted that for CD subjects, only adolescents with “pure” (i.e., no any other comorbidities) CD were considered, largely due to the significant functional differences between “pure” CD patients and healthy controls [4, 35–37]. Furthermore because many neurological and psychiatric disorders differ substantially between males and females [38, 39], only male adolescents with pure CD were included in this study. We believed that the novel findings in CD patients will yield new evidence to validate the dysfunctional network hypothesis and provide new insights into its basic neural mechanism.

## Materials and Methods

### Subjects

Eighteen right-handed male adolescents with pure CD (aged 15–17 years) were recruited from the Hunan Province Youth Detention Center in the People’s Republic of China. In addition, eighteen precisely age-, handedness- and sex-matched TD controls were recruited from the local communities in Changsha City, Hunan Province. All the participants as well as their parents or caregivers provided written informed consents before the experiment. The present study was approved by the Biomedical Ethics Board of the Second Xiangya Hospital, Central South University, China and the Biomedical Ethics Board with Faculty of Health Sciences at the University of Macau (Macao SAR, China).

Participants were assessed for their present and lifetime psychiatric diagnoses using the Chinese version of the Schedule for Affective Disorder and Schizophrenia for School-Age Children-Present and Lifetime (K-SADS-PL) [40–42], in accordance with the DSM-5 criteria [1]. Participants who met the K-SADS-PL criteria for CD were included in this study, and any subject who had comorbid conditions with other current and lifetime psychiatric problems, such as attention deficit/hyperactivity disorder, obsessive-compulsive disorder, and substance abuse were excluded from the present study. In addition, participants were screened to make sure that they had no reported neurological diseases, including epilepsy, chronic pain, loss of consciousness, confusion and traumatic brain damage and that they had not taken medicine for at least 3 months prior to participating in this study.

### fMRI data collection

During the resting-state scans, all the subjects were instructed to rest quietly with their eyes closed and relaxed without thinking about anything in particular or falling asleep. Data acquisition was performed using a 3.0-Tesla MR scanner (Siemens Allegra), which was located at the Magnetic Resonance Center of Hunan Provincial People’s Hospital; the overall process lasted 6 minutes and 36 seconds. An eight-channel head coil was used for all MRI scans and a tight but comfortable foam padding was utilized to minimize the head motion for all subjects. Functional images were generated using single-shot, gradient-recalled echo-planar imaging (EPI) sequences according to the following parameters: TR = 3,000 ms, TE = 30 ms, and flip angle = 90°. Thirty-six contiguous axial slices (field of view = 256 mm × 256 mm, in-plane matrix = 64 × 64, slice thickness = 3 mm, no gap), aligned along the anterior commissure-posterior commissure line, were acquired. For each subject, a total of 100 volumes were acquired for image reconstruction.

### fMRI data preprocessing

Resting-state fMRI data were preprocessed using Matlab with the Statistical Parametric Mapping software package SPM8 (http://www.fil.ion.ucl.ac.uk/spm). The first ten volumes were removed from each subject to allow for the magnetization equilibrium and saturation effects. Afterwards, the remaining 90 volumes were first slice-timing corrected and then registered to the first of the remaining volumes for head motion correction. No subjects were excluded from further analysis because their translational or rotational parameters did not exceeded ± 1 mm or ± 1°. Further preprocessing included spatial normalization into the standard Montreal Neurological Institute (MNI) space with a resampling voxel size of 3 × 3 × 3 mm^3^ and spatial smoothing by convolution with an isotropic Gaussian kernel of 8 mm full width at half maximum.

### ICA and identification of RSNs

fMRI data (data in **S1–S11 Appendixes**) of all the subjects, including the CD and TD groups, were processed together to construct the RSNs based on a group-level spatial ICA. Group ICA was performed to decompose the data into independent components (ICs) using the GIFT toolbox (http://icatb.sourceforge.net/, version v3.0a) [43]. The preliminary estimation of the dimension of all subjects was conducted using the minimum description length criterion [44], and eventually, 30 ICs were determined. Data were then reduced following two reduction steps. Firstly, all the data from each subject were reduced. Then, the compressed datasets of each subject were concatenated into one group, and this aggregate dataset was reduced again to 30 components using principal component analysis, followed by an IC estimation using the Infomax algorithm [45, 46]. In the final stage, the generated spatial maps and associated time courses corresponding to the ICs for each subject were back-reconstructed by a dual-regression method [47–49]. The intensity values for each subject-specific spatial map were transformed to z-scores, and a high z-score within the spatial maps indicated a greater contribution of a given voxel to its related IC time course [50, 51]. Because ICA was widely recognized to have the capability to extract a specific pattern of spontaneous brain activity, we could directly use z-scores as measurements of functional connectivity within the brain networks [30, 52].

To select valid RSNs, the ICs were visually inspected to identify the obvious artifacts. Then, the spatial multiple regression was performed for each IC with *a priori* probabilistic templates of gray matter, white matter and cerebrospinal fluid provided in SPM8. ICs that showed high associations with white matter or cerebrospinal fluid and low correlations with gray matter were excluded from further analysis. Next, the spatial maps of the 30 ICs were recognized according to the fMRI networks during the resting state with the largest spatial correlations [53–55], in combination with RSN templates [51, 56, 57], leaving a total of nine representative RSNs for further analysis.

### Statistical analysis

The spatial maps for each of the RSNs extracted from ICA were gathered using one-sample *t*-tests for both the CD and TD group, with a threshold of *p* < 0.05 in SPM8 [false discovery rate (FDR) corrected] [58]. To demonstrate the differences within each network between the CD and TD groups, each of the RSNs was performed with two-sample *t*-tests. Group comparisons were restricted to the voxels within corresponding RSNs. Two-sample *t*-tests were conducted using the mask for each of the RSNs. The statistical maps of RSNs based on the results of one-sample *t*-tests for each subject of both the CD and TD group were binarized and combined together using the term “AND” to create the mask used for two-sample *t*-tests. The mask was only used to determine the brain regions of the comparison between the two groups. Data were corrected with a threshold of *p <* 0.05, which was set at a threshold of *p <* 0.01 combined with a cluster size of at least 52 voxels using the AlphaSim program based on the REST toolbox (http://www.restfmri.net/forum/REST_V1.8) based on the Monte Carlo simulation [59] (for more detailed information, please see http://afni.nimh.nih.gov/pub/dist/doc/manual/AlphaSim.pdf).

## Results

### Demographic features

Demographic characteristics of the CD and TD group are presented in **Table 1**. There were no significant differences in age and educational level between the two groups. However, we did find significant differences in psychiatric scores between the CD patients and healthy controls (*p* < 0.001).

**Table 1:** Demographic features of CD patients and TD subjects.

Characteristics	CD (n = 18)	TD (n = 18)	CD *vs*. TD
	M ± SD	M ± SD	*p* value
**Age (yrs)**	16.1 ± 0.5	15.9 ± 0.3	0.27
**Education (yrs)**	9.4 ± 2.0	9.2 ± 1.9	0.47
**Mother’s education (yrs)**	8.2 ± 4.1	10.1 ± 3.5	0.13
**Father’s education (yrs)**	8.8 ± 2.6	10.4 ± 2.2	0.07

CD, Conduct Disorder; TD, Typically Developing; M, Mean Score; SD, Standard Deviation; yrs, years; vs., versus.

### RSNs in the CD and TD groups

**Fig 1:**
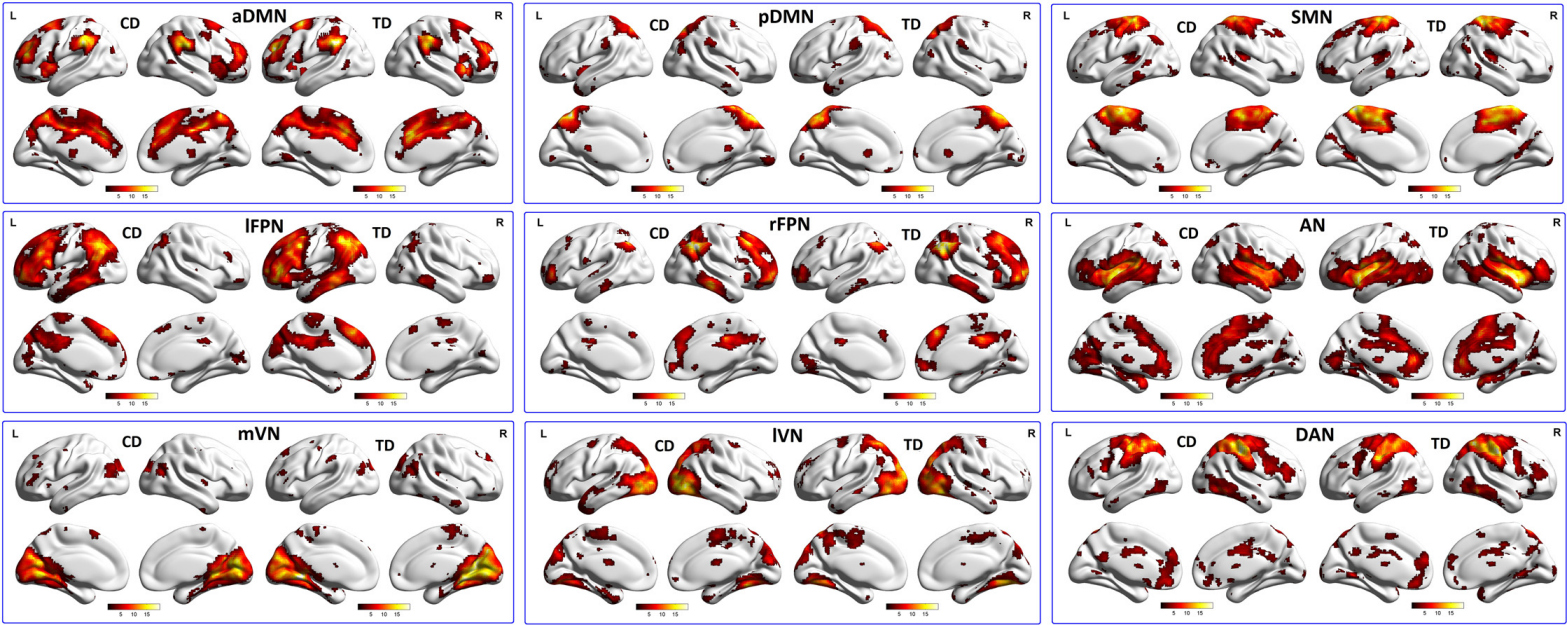
Results of one-sample *t*-tests of nine RSNs in the CD and TD groups, including pDMN, aDMN, lFPN, SMN, AN, rFPN, lVN, mVN, and DAN. Lateral and medial views of the left hemisphere and lateral and medial views of the right hemisphere for each group, as shown by BrainNet Viewer. The color scale represents *T* values with a range of 0.01~20 in each RSN (*p* < 0.05, FDR corrected). L, Left; R, Right; CD, Conduct Disorder; TD, Typically Developing; RSN, resting-state network. pDMN, posterior Default Mode Network; aDMN, anterior Default Mode Network; lFPN, left Frontal-Parietal Network; SMN, Somato-Motor Network; AN, Auditory Network; rFPN, right Frontal-Parietal Network; lVN, lateral Visual Network; mVN, medial Visual Network; DAN, Dorsal Attention Networks.

As shown in **Fig 1**, nine spatial maps of these RSNs were revealed by ICA, in which multiple cortical regions were identified to be engaged in the RSNs. It was observed from **Fig 1**that the generated RSNs included: 1) the posterior default mode network (pDMN), which was represented by IC and contained the posterior cingulate cortex, precuneus, angular and inferior parietal lobe; 2) the anterior default mode network (aDMN), which was constructed from IC related to the medial/superior frontal cortex and anterior cingulate cortex; 3) the left frontal-parietal network (lFPN), which was selected from IC that involved the left hemisphere of the dorsal lateral prefrontal cortex and posterior parietal cortex; 4) the right frontal-parietal network (rFPN), which was identified from IC including the corresponding areas of the right hemisphere; 5) the somato-motor network (SMN), which was constructed from IC encompassing the pre- and post-central gyrus and supplementary motor area (SMA); 6) the auditory network (AN), which was selected from IC mainly related to the bilateral middle and superior temporal gyrus; 7) the lateral visual network (lVN), which was recognized from IC whose key regions were the inferior and middle occipital gyrus along with the superior parietal gyrus; 8) the medial visual network (mVN), which was identified from IC predominately relevant to the superior occipital gyrus, the temporal-occipital regions and fusiform gyrus; and 9) the dorsal attention network (DAN), which was identified from IC and primarily involved the middle and superior frontal gyrus, superior parietal gyrus, intraparietal sulcus, and frontal eye fields.

### Altered RSNs in the CD group

**Fig 2:**
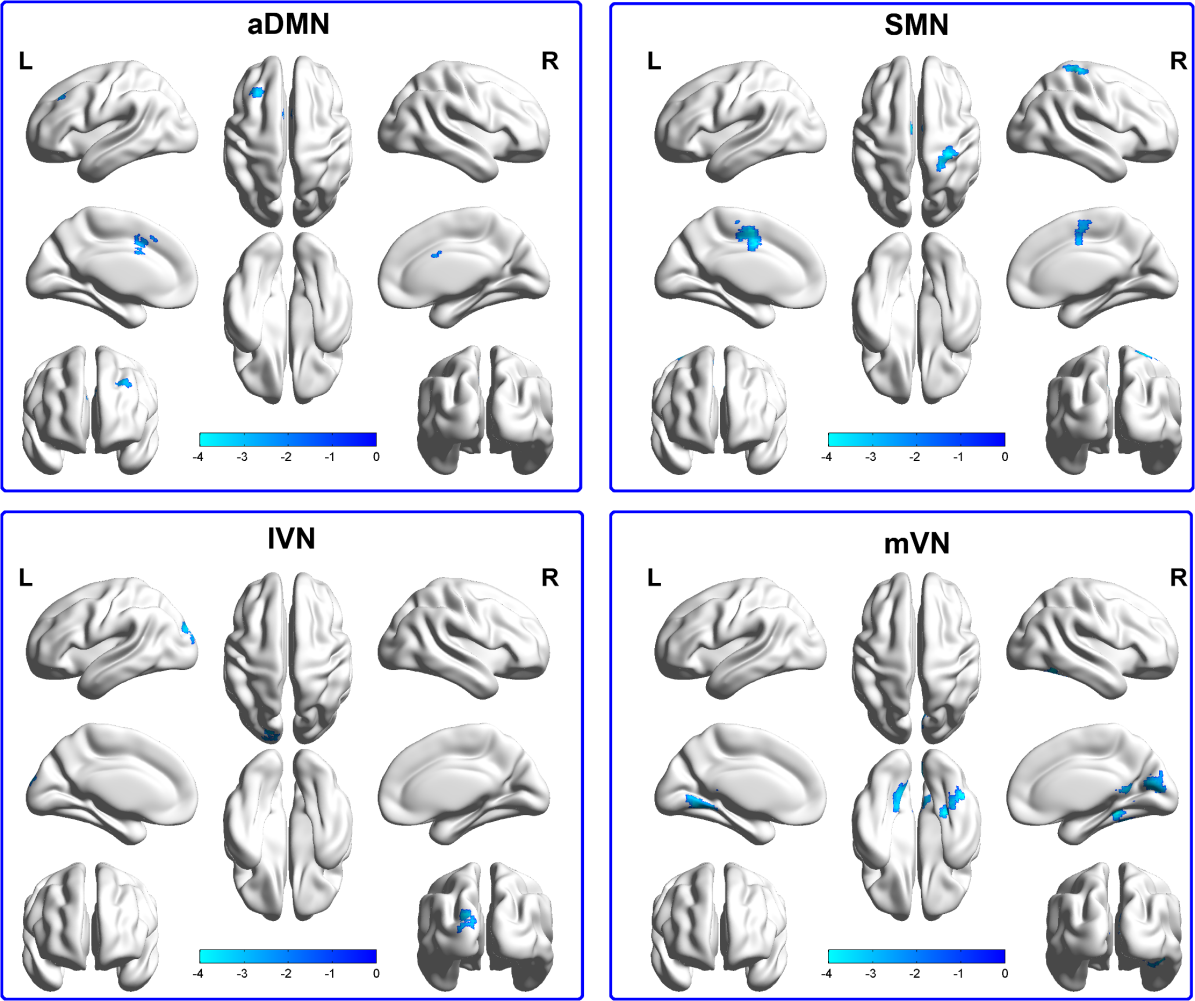
Brain regions with significant differences for four RSNs in the CD group vs. the TD group (*p* < 0.05, AlphaSim corrected). Cool colors indicated the decreased functional connectivity in the CD group compared with the TD group, as shown by the BrainNet Viewer. L, Left; R, Right; aDMN, anterior Default Mode Network; SMN, Somato-Motor Network; lVN, lateral Visual Network; mVN, medial Visual Network.

The results of the two-sample *t*-tests in **Fig 2**and **Table 2**illustrated that the functional connectivity in RSNs was significantly different between the CD and TD groups in several brain regions. In comparison with the TD group, the CD group showed decreased functional connectivity in the aDMN, including the left middle frontal gyrus; the SMN, including the bilateral SMA and right postcentral gyrus; the lVN, including the left superior occipital gyrus; and the mVN, including the right fusiform gyrus, left lingual gyrus and right calcarine (*p <* 0.05, AlphaSim corrected). In particular, **Table 2**lists the brain regions with abnormal functional connectivity for each of the RSNs.

**Table 2:** Decreased functional connectivity of four RSNs in the CD group compared with the TD group.

RSNs	Brain areas	MNI coordinates			*T* value	Voxels
		X	Y	Z		
**SMN**	R-SMA	3	-6	51	-3.5	66
	L-SMA	-9	-12	51	-3.48	56
	R-PG	27	-39	69	-4.0189	71
**lVN**	L-SOG	-15	-93	15	-3.42	57
**mVN**	R-FG	27	-39	-12	-4.3824	80
	L-LG	-18	-57	-9	-3.6171	100
	R-CAL	12	-75	15	-3.58	77
**aDMN**	L-MFG	-21	39	24	-3.954	83

CD, Conduct Disorder; TD, Typically Developing; RSN, Resting-State Network; MNI, Montreal Neurologic Institute; SMN, Somato-Motor Network; lVN, lateral Visual Network; mVN, medial Visual Network; aDMN, anterior Default Mode Network; SMA, Supplementary Motor Area; PG, Postcentral Gyrus; SOG, Superior Occipital Gyrus; FG, Fusiform Gyrus; LG, Lingual Gyrus; CAL, Calcarine; MFG, Middle Frontal Gyrus; L, Left; R, Right. All the coordinates are denoted by MNI space coordinates. Negative *T* value represents the statistical value of a peak voxel, which indicates the decreased functional connectivity in the CD group.

## Discussion

To the best of our knowledge, this is the first study to investigate whether the intrinsic functional connectivity might be altered in male adolescents with pure CD using rs-fcfMRI. In this work, nine meaningful RSNs were identified and extracted using group ICA to show the differences in the neural features between the CD and TD groups. Compared to the findings of the TD group, reduced functional connectivity was observed in four representative RSNs, including the SMN (bilateral SMA and right postcentral gyrus), the lVN (left superior occipital gyrus), the mVN (right fusiform, left lingual gyrus and right calcarine), and the aDMN (left middle frontal gyrus). In addition, the DMN plays an essential role in maintaining the baseline activities in brain cognitive functions associated with intrinsic processing, episodic memory, environmental monitoring and future thought [60, 61]. In this study, the DMN, including the medial prefrontal gyrus, posterior cingulate/precuneus, middle temporal lobe, bilateral inferior parietal gyrus, and angular gyrus, was further divided into the aDMN and pDMN [62]. Interestingly, we found that the subjects in the CD group showed major differences in brain networks compared to the healthy controls, where decreased functional connectivity in the aDMN, including the left middle frontal gyrus, was revealed in CD patients. Our findings were in good agreement with those of previous studies, which showed that individuals with CD exhibited a failure of inhibition and restraint due to functional and structural anomalies in brain regions associated with social cognition and introspective processes [63, 64]. These abnormalities can be detected by assessing the brain activity of DMN, as the brain regions were more active during the resting state than during the performance of a wide range of cognitive and attention tasks [65]. Moreover, Dalwani et al. [66] also indicated decreased activity in the superior and middle frontal gyrus in CD and substance use disorder (SUD) patients when compared to controls, suggesting that the atypical activity of CD and SUD adolescents was related to brain areas involved in introspective processing. Furthermore, brain structural studies reported that patients with CD showed reductions in the grey matter volume in the middle frontal gyrus relative to healthy controls [67]. Impairment of prefrontal areas could cause the aberrant development of social and moral behavior, which could lead to CD symptoms [68]. Lesion studies have demonstrated that the superior frontal gyrus plays an important role in higher levels of cognitive processing, particularly in working memory [69]. Likewise, our results suggested that the functional deficits in the middle frontal gyrus and superior frontal gyrus were correlated to the CD patients’ inability to engage effectively in higher-order cognitive function and introspective processes.

More interestingly, compared with the TD group, the CD group exhibited significantly reduced functional connectivity in the lVN, including the left superior occipital gyrus; the mVN, including the right fusiform gyrus, left lingual gyrus and right calcarine; and the SMN, including the bilateral SMA and right postcentral gyrus. The VN, AN, and SMN are considered to comprise the low-level perceptual systems, which contribute to low-order cognitive processing [70]. For the subjects in the CD group, the present study showed a regional decrease in the functional connectivity in SMA, which is involved in the processes of interoceptive, autonomic, homeostatic, and cognitive information of personal relevance [71, 72]. The present results also showed good agreement with previous observations [73–75]. For instance, in a task-driven fMRI study, Decety et al. found that when viewing a situation in which a person intentionally inflicted pain on others, CD patients showed increased activation in the SMA than the controls, illustrating that CD patients react to pain to a larger extent than the controls [73]. Most notably, a wide variety of structural neuroimaging studies have provided evidence of structural abnormalities in brain regions of VN. For example, in a voxel-based morphometry (VBM) study, Huebner et al. demonstrated that boys with CD had smaller gray matter volume in the right fusiform gyrus and middle occipital gyrus compared to the controls [74]. Another VBM study indicated that the combined CD group (early-onset plus disorder-onset) also had reduced regional gray matter volume in the left superior occipital cortex and fusiform gyrus [75]. Furthermore, Rubia et al. proposed that CD patients showed decreased activation in a cluster, including occipital regions relative to the healthy comparison subjects during sustained attention [4]. In conclusion, the aberrant functional connectivity in SMN, lVN and mVN identified in the present study may provide further evidence for understanding the impairments of socio-emotional processing in CD patients during the resting state.

## Limitations

Several potential limitations of this study should be mentioned. First, the relatively small sample size and some important clinical measures, such as duration and severity of disease, that were not recorded for CD patients might affect the accuracy of statistical analysis and the brain mapping results. Secondly, we did not detect physiologic signals, such as the heart and respiration rate, during the scan, and the relatively long TR (3s) of the MRI sequences in this study could interfere with the low-frequency oscillation during the resting-state signal detection [76]. Furthermore, some methodological limitations related to ICA should also be considered; for example, selecting the target ICs is a common issue for data-driven approaches. In numerous studies, the number and the spatial pattern of the RSNs detected by ICA could vary [26, 30, 77, 78]. Therefore, the networks in this study may or may not be identified in a single IC. Finally, the neurophysiological mechanisms of RSNs during the resting state are still ill-defined compared to the task-related fMRI studies [79]. At this point, using specific task paradigms to directly investigate the perceptual impairments in CD patients should be worth considering in the future.

## Conclusions

In summary, selective impairments of resting-state intrinsic functional connectivity were observed in male adolescents with pure CD. In particular, we found that aDMN, lVN, mVN and SMN showed decreased functional connectivity when compared to those from TD healthy controls, whereas pDMN, lFPN, rFPN, DAN and AN were unaffected. Diminished functional connectivity of brain regions in RSNs would facilitate our understanding of the neural mechanisms involved in emotion modulation, social cognition and perception regulation in CD patients. Our findings provide novel evidence of the relationship between the low-level perceptual networks and high-order cognitive networks in CD patients. Further efforts to reveal the more refined neurophysiological processes of CD should benefit from combinations with other neuroimaging modalities, such as electroencephalography (EEG) and functional near-infrared spectroscopy (fNIRS), or from integration with specific task-driven studies and dynamic connectivity analysis methods.

## Supporting Information

S1 Appendix The 4 dataset of conduct disorder patients from 1 to 4 used in this study. (ZIP)

S2 Appendix The 4 dataset of conduct disorder patients from 5 to 8 used in this study. (ZIP)

S3 Appendix The 4 dataset of conduct disorder patients from 9 to 12 used in this study. (ZIP)

S4 Appendix The 3 dataset of conduct disorder patients from 13 to 15 used in this study. (ZIP)

S5 Appendix The 3 dataset of conduct disorder patients from 16 to 18 used in this study. (ZIP)

S6 Appendix The 3 dataset of typically developing subjects from 1 to 3 used in this study. (ZIP)

S7 Appendix The 3 dataset of typically developing subjects from 4 to 6 used in this study. (ZIP)

S8 Appendix The 3 dataset of typically developing subjects from 7 to 9 used in this study. (ZIP)

S9 Appendix The 3 dataset of typically developing subjects from 10 to 12 used in this study. (ZIP)

S10 Appendix The 3 dataset of typically developing subjects from 13 to 15 used in this study. (ZIP)

S11 Appendix The 3 dataset of typically developing subjects from 16 to 18 used in this study. (ZIP)
